# Gene Expression and Phenotypic Assessment of Egg Quality across Developmental Stages of Atlantic Cod throughout the Spawning Season

**DOI:** 10.3390/ijms25137488

**Published:** 2024-07-08

**Authors:** María Fernández Míguez, Pablo Presa, Velmurugu Puvanendran, Helge Tveiten, Øyvind J. Hansen, Montse Pérez

**Affiliations:** 1 Department of Biological Sciences, University of Bergen, 5006 Bergen, Norway; 2 Laboratory of Marine Genetic Resources, ReXenMar, CIM, Universidade de Vigo, 36310 Vigo, Spain; pressa@uvigo.gal; 3 AQUACOV, Centro Oceanográfico de Vigo, Instituto Español de Oceanografía (IEO, CSIC), 36202 Vigo, Spain; montse.perez@ieo.csic.es; 4 Department of Production Biology, Centre for Marine Aquaculture, Nofima AS, 9291 Tromsø, Norway; velmurugu.puvanendran@nofima.no (V.P.); oyvind.j.hansen@nofima.no (Ø.J.H.); 5 Faculty of Biosciences, Fisheries and Economics, Norwegian College of Fishery Science, The Arctic University of Norway (UiT), 9019 Tromsø, Norway; helge.tveiten@uit.no

**Keywords:** Atlantic cod, *Gadus morhua*, developmental stages, egg quality, gene expression, quantitative PCR

## Abstract

Egg quality in fishes is commonly determined by fertilisation success and cleavage patterns as a phenotypic outcome of underlying regulatory mechanisms. Although these phenotypic estimators of egg quality are useful in farming conditions, these “good quality” egg batches do not always translate to good larval growth and survival. The identification of genes involved in embryonic development may help find links between genetic factors of maternal origin and egg quality. Herein, the relative expression of seven stage-specific developmental genes of Atlantic cod was analysed using quantitative PCR to understand the function during embryogenesis and its relationship with egg quality. Genes *ccnb2* and *pvalb1* showed significant differential expression between developmental stages and significant upregulation from blastula and somite stages, respectively. The comparison of spawning batches showed that the relative gene expression of genes *ccnb2*, *acta*, *tnnt3* and *pvalb1* was significantly higher from the middle of the spawning season where phenotypic quality estimators establish the best egg quality. Moreover, a positive significant correlation was observed between quality estimators based on egg morphology and the genetic expression of genes *acta* and *acta1* during somitogenesis. This study suggests that the combination of quality estimators, genetics and batch timing could help optimise reproductive protocols for commercial stocks of Atlantic cod.

## 1. Introduction

Atlantic cod (*Gadus morhua* L.) is a key trophic species from the North Atlantic and a potential species for aquaculture in this region including Norway. Since the 1980s, farming cod has been of interest to many North Atlantic countries, especially in Norway, the UK, Canada, Iceland, and the Faroe Islands. However, it was a boom-and-bust activity until now. Interest in farming cod was highest in the 2000s, producing 20,000 t in 2009 but decreasing to less than 2000 t in 2014, either due to biological reasons such as a high and unpredictable mortality rate in early life stages and poor quality of the juveniles, or to an economic meltdown in Europe in late 2000s with increasing wild fisheries quotas and decreasing market prices [1,2]. Despite the reduced interest in cod farming since 2010, research to solve biological issues, especially improving the quality of the juveniles and growth, continued in Norway thanks to the National Cod Breeding Program (NCBP) funded by the Norwegian government. Since 2018, interest in cod farming reignited due to higher market prices. Despite the significant progress made in its culture (i.e., cost-efficient feeds, disease control, improved juvenile quality and growth) as well as the life cycle closure in captivity [3,4], further development requires the production of good quality eggs needs more research to further improve the cod aquaculture. For instance, cod fertilisation success can vary from <10% in poor batches to >90% in good batches [5] and higher mortality during egg incubation and larval rearing is often significantly higher in eggs and larvae originating from farmed broodstock than from wild fish indicates that further research is needed in broodstock feed development and husbandry [6].

Knowledge of mechanisms controlling egg quality is necessary to improve the production of newly domesticated fish species [7]. Atlantic cod produces several egg batches over a prolonged period, i.e., from winter to late spring, and shows a decreasing egg quality and size towards the end of the spawning season [8,9]. Egg quality is commonly defined as the ability of the egg to be fertilised and subsequently develop into a normal embryo [10] as well as the potential to hatch into a viable larva [11]. It is generally assumed that good quality eggs would exhibit higher fertilisation, symmetrical embryonic cleavage, and low mortality during incubation, with larvae performing optimally at first feeding [12]. However, such “good quality” eggs may have lower hatching rates, indicating the partiality of classical quality determinants to characterise egg quality [13,14] as it can be affected by many environmental and biological factors at various stages of the oogenesis [10,15]. Several studies showed that yolk composition has a significant effect on embryonic development [16,17]. Additionally, maternal mRNAs that accumulate in the oocyte are essential in governing early embryonic development [18,19], i.e., from fertilisation to mid-blastula when the zygotic genome takes over the control of embryonic development [20,21]. In Atlantic cod, a considerable amount of information is available on the influence of incubation temperature on maternal factors [22], characterisation of untranslated regions of maternal genes [23] and transcriptome profiling of maternal and zygotic genes [20,24,25,26]. However, little is known about the putative influence of genetic factors on egg quality. Therefore, the characterisation of genetic factors explaining a part of the variability in egg quality among egg batches could help diminish the incidence of malformations and mortality rates during early life stages [24].

Egg quality measures in cod are mainly based on egg morphology, biochemical composition, fertilisation success and early cleavage patterns [27,28]. The latter two are generally applied in cod breeding facilities as standard estimators of egg quality to select progeny batches that are to be used for the first feeding stage [28]. Although those estimators provide a reasonable indication of quality, especially the embryonic cleavage pattern [28], knowledge of the molecular basis of embryo quality would facilitate selective practices. For instance, several egg-quality related studies used expressed sequence tags (ESTs), microarrays and whole genome sequencing in model species such as zebrafish and pufferfish [29,30]. Also, sequencing efforts have produced a large amount of data from farmed fish, especially ESTs and microarrays from Atlantic salmon [31,32,33], Atlantic halibut [34], Atlantic Bluefin tuna [35] and other species. In the Atlantic cod, about 150,000 ESTs have been reported [36] along with an RNA deep sequencing development [25,37,38,39]. These studies highlight the growing importance of understanding the role of genetic factors regulating embryonic development to minimise both early egg batch losses and rearing poor quality larvae in Atlantic cod farming.

The present study aimed to test whether standard egg quality measures such as fertilisation success and normal cleavage along the spawning season were correlated with the up/downregulation of some stage-specific embryonic genes. We hypothesise that under such a correlation, knowledge of the expression profiles of potential broodfish would help as quality predictors of their offspring.

## 2. Results

### 2.1. Egg and Larvae Characteristics

**Figure 1:**
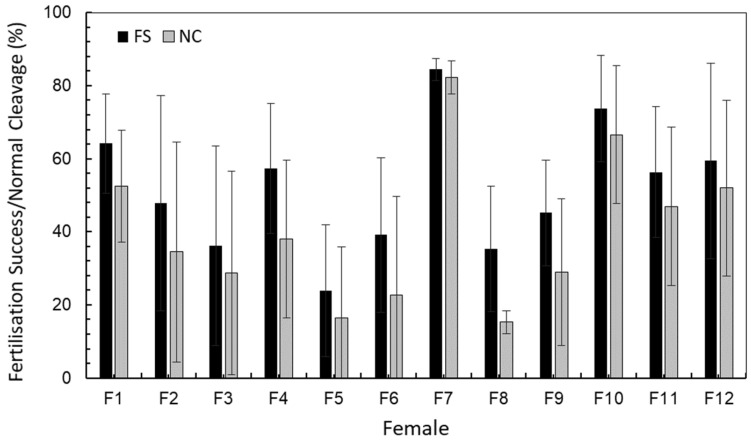
Fertilisation success (FS %) and normal cleavage (NC %) variation among cod females, as averaged (±SD) among spawning batches within females. See Table 1 for ANOVA analysis between females and Tukey post hoc test.

Seasonal average of fertilisation success (FS) and normal cleavage (NC) differed significantly among females (FS, alpha = 0.981; NC, alpha = 0.996; see Tukey test in Table 1). Mean values of female 7 reached 80% quality as a seasonal average (Table 1) and other females exceeded the standards in some batches (Figure S1). Mean values of quality estimators among females fluctuated 20–80% (Figure 1), besides patent differences among spawning batches (S1–S7) within females (Table 1).

No significant differences were observed in dead egg volume among batches within females (ANOVA, *p* = 0.859), only female 4 differed among females (Tukey post hoc F = 3.890, F_critical = 1.494, *p* = 2.7 × 10^−4^, Table S1). The mortality rate (MR) differed significantly among females (ANOVA, *p* = 0.0002) and ranged from 2.34% in female 7 to 9.37% in female 4 which had the highest spawn and dead volume (Table S1). Pearson correlation and linear regression of standard egg quality estimators showed a significant positive correlation between FS and NC (Pearson R = 0.912; *p* < 0.001, Figure S2) and a negative correlation of these two parameters to mortality rate (MR) estimation (R = −0.37; *p* = 0.001, Figure S2), but no correlation to larvae length.

Spawning properties and egg quality indicators of five females whose offspring were transferred to first feeding tanks showed no differences among females on larval length (Figure S3). Only female 5 differed significantly from the rest of the females at t30 days post-hatch (Tukey post-hoc *p* = 0.01, Table S2) despite showing low FS and NC values at the egg stage (Table 2). The resulting increase in larval length in this female may be due to the density differences, i.e., poor survival leads to the opportunity to use the resources of the remnant larvae. Wet weight of larvae did not differ among females. Larvae from females 5 and 11 showed the highest and the lowest wet weight, respectively, but t90 weight variation did not differ among females, i.e., (*p* = 0.126, Table S3).

### 2.2. Gene Expression

Seven stage-specific genes (see stages in Table 3) were selected for qPCR analyses based on their optimal efficiency values (E = 83–101; Table 3, Figure S4). Gene *ccnb2* was analysed on unfertilised eggs while the remaining genes were amplified after fertilisation until larvae hatched. The un-normalised mean CT levels of target genes varied from CT = 24 for *pvalb1* to CT = 28 for *myhc* (Figure S5a). The most stable target and reference genes exhibiting the lowest CT variation across assays were *acta* and *rps9*, respectively (Figure S5a,b). The *arp* reference gene had the highest standard deviation, as previously observed after normalisation analyses (Table S4).

**Figure 2:**
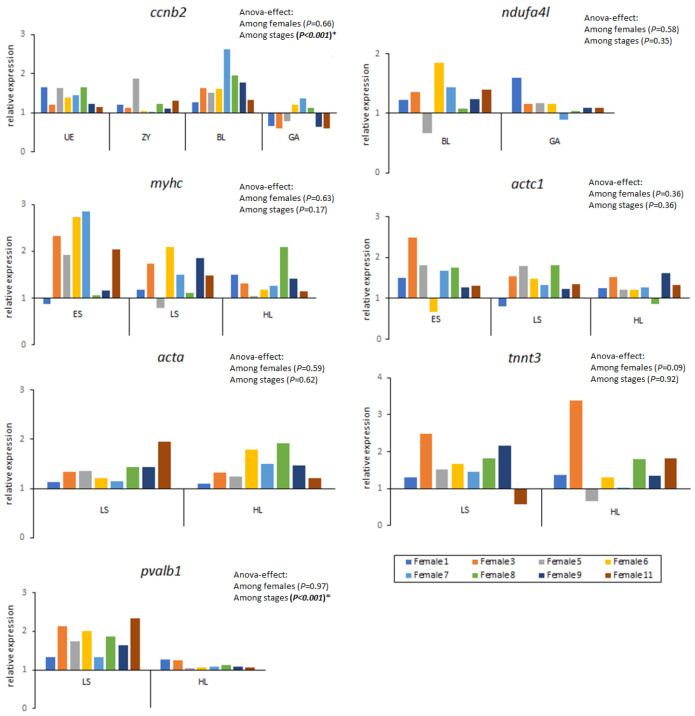
Relative expression of seven target genes (*ccnb2*, cyclin B2; *ndufa4l*, NADH ubiquinone reductase 4l; *myhc*, myosin; *actc1*, actin alpha cardiac muscle 1; *acta*, actin alpha skeletal muscle; *tnnt3*, troponin T skeletal; *pvalb1*, parvalbumin 1) during cod developmental stages: unfertilised egg (UE), zygote (ZY), blastula (BL), gastrula (GA), early somitogenesis (ES), late somitogenesis (LS) and newly hatched larva (HL). Data from each developmental stage are presented as the average of all spawn batches analysed. Global ANOVA significant differences among females and among stages (pairwise post hoc probabilities after Tukey’s test are given in Results). *, *p* < 0.001.

Most targeted genes were not differently expressed among developmental stages, only *ccnb2* and *pvalb1* showed a significant effect of stage. Gene *ccnb2* involved in cell division was expressed at an early stage and significantly upregulated during the blastula (BL) stage (Figure 2). In addition, gene *pvalb1* related to calcium signalling was upregulated during the late somitogenesis stage (LS) in all females but significantly decreased in hatching larvae (HL) (Figure 2). Relative expression of the *myhc* and *actc1* genes was high during ES and slowly reduced until HL, but not significant. Relative expression of the *acta* gene was constant among batches and among developmental stages during both late somite (LS) and HL while expression of *tnnt3* decreased at HL.

**Figure 3:**
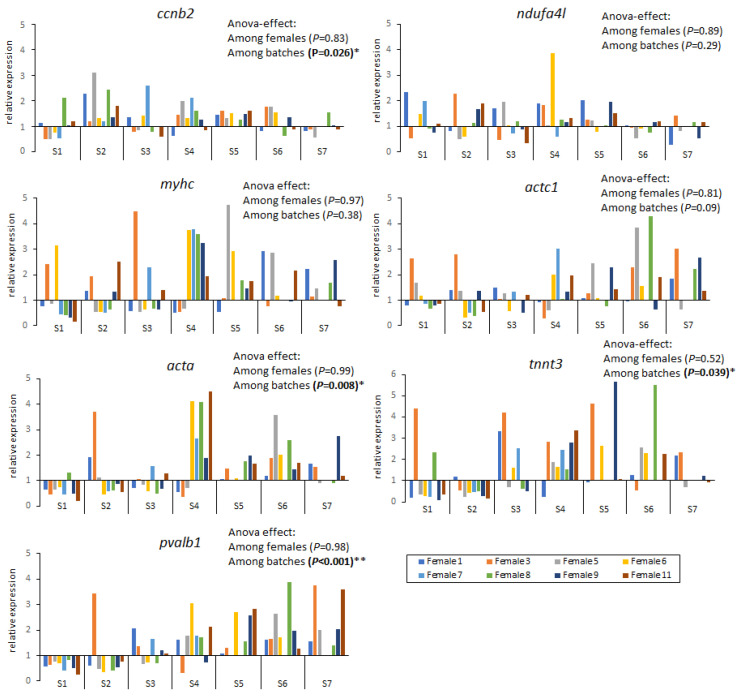
Relative expression of seven target genes (*ccnb2*, cyclin B2; *ndufa4l*, NADH ubiquinone reductase 4l; *myhc*, myosin; *actc1*, actin alpha cardiac muscle 1; *acta*, actin alpha skeletal muscle; *tnnt3*, troponin T skeletal; *pvalb1*, parvalbumin 1) in seven spawning batches (S1–S7) from eight cod females (F1, F3, F5, F6, F7, F8, F9 and F11). Data from each batch spawn are presented as the average of all developmental stages analysed (see Table 3). Pairwise post hoc probabilities after Tukey’s test *, *p* < 0.05; **, *p* < 0.001.

On the other hand, the variation in gene expression showed no significant differences among females but there were significant differences among spawning batches (within a female) (Figure 3). Genes *myhc*, *acta1* and *ndufa4l* exhibited a similar expression along the spawning season, while genes *ccnb2*, *acta*, *tnnt3* and *pvalb1* were differently expressed among spawning batches and were upregulated after spawning batch 4 (see *p* values in Figure 3).

### 2.3. Molecular and Phenotypic Data Correlation

A negative correlation was found between the mortality rate and the other two quality indicators based on morphology, fertilisation success and normal cleavage but non-significant (Table 4). A positive significant correlation was observed, using average data from all females, between phenotypic estimators of egg quality (FS and NC) and the genetic expression of genes *acta* and *acta1*. Gene expressions of *acta* and *actc1* genes were also positively correlated with those of genes *myhc* and *pvalb*, while the tnnt3 gene was positively correlated to *pvalb1* (Table 4). 

As per female expression, genes *acta*, *acta1* and *pvalb1* were significantly positively correlated with fertilisation success, while genes *acta*, *actc1*, *ccnb2*, *ndufa4l* and *tnnt3* with normal cleavage (see * in Table 5). Mortality rate showed a significant negative correlation with gene *myhc* and a positive correlation with *ndufa4l* (Table 5). After statistical analysis of egg quality indicators, females 3, 5 and 8 would be considered “poor-quality” while females 1, 7 and 10 “good-quality”.

## 3. Discussion

Good quality eggs have been defined as those exhibiting low mortalities during embryonic stages, hatching and first feeding [12]. Although a broodstock can produce large quantities of eggs, their quality often varies during the spawning season, e.g., gilthead seabream, *Sparus aurata* [7], red seabream, *Pagrus major* [42], turbot, *Scophthalmus maximus* [43], Atlantic cod, *Gadus morhua* [44], Atlantic halibut, *Hippoglossus hippoglossus* [45], Atlantic wolffish, *Anarhichas lupus* [46]. Egg quality can rapidly decrease for batch spawning species during a spawning season and could be affected by species-specific differences in the time scale of overripening and the ageing process when eggs are retained within the broodfish after ovulation [45]. Thus, it is interesting to determine the best timing for collecting high-quality eggs as well as to find reliable methods to assess the viability of fertilised eggs in their early development.

### 3.1. Quality Estimations Based on Morphology

Egg batches obtained during the whole spawning season of Atlantic cod showed differences in quality and embryonic developmental competence among females. Such differences were compared between developmental stages as well as between spawning batches and were correlated with the relative expression of genes involved in zygotic formation and embryogenesis. Results indicated that fertilisation success and normal cleavage were positively correlated with each other as previously reported [28] but only marginally negatively with early embryonic mortality rate. This latter differed significantly among females, i.e., from 2.34% in the best female 7 to 9.37% in female 4 irrespective of their spawning volume. Traditionally, the egg quality of cultured marine fish species is measured at early stages (unfertilised or freshly fertilised eggs) based on morphological parameters and the presence of over-ripped eggs [47]. Those estimators not only distinguished between viable and non-viable eggs but also allowed us to estimate the percentage of eggs completing the development. Common variables to check egg quality are egg transparency, i.e., poor quality eggs are normally opaque [44,48,49]; egg buoyancy, i.e., poor quality eggs normally sink to the bottom of a tank [50]; location of the lipid droplet around the equatorial area [49,50]; or egg size, i.e., provides an estimate of parental investment in offspring [45]. We have also used parameters such as colour, transparency and buoyancy in unfertilised eggs to identify good/bad quality eggs but did not discard any due to the experimental setup of this study.

Although it cannot be generalised, fertilisation success has been reported to decrease with the advancement of the spawning season, e.g., Senegalese sole [51] and Atlantic cod [9]. Other gadoid species such as the European hake show at least two spawning peaks throughout the year in their southern stock and a direct influence of female size on offspring quality [52]. In our study, this generalisation was not evident in most of the females and the FS and NC varied among batches within a female. Lower FS and/or NC may not indicate bad quality eggs because the sperm quality can also affect the FS and subsequently the NC [53]. Unfortunately, we could not determine the quality of the sperm used in our study to verify this. The fertilisation success is estimated as the percentage of fertilised eggs, expressing the successful encounter between egg and sperm. Although commonly used quality criteria in both marine and freshwater species [54,55,56], inconsistent correlations with egg and larvae survival rates have been reported in some marine species [50], which could be related to the different egg division stages employed to assess fertilisation success [47,54].

Alternatively, hatching success is suggested to be better correlated to normal cell symmetry at early stages of cleavage (normal blastomeres) measured within 24 h of fertilisation than fertilisation success in marine fishes, e.g., Atlantic cod [44,57], halibut [58], wolffish [46] and turbot [55]. Further, abnormal cleavage patterns are correlated with early egg mortality, low hatching success, and larval abnormalities in Atlantic cod [27,59,60]. In our study, fertilisation success and early cleavage have shown a positive correlation while showing a negative correlation of these two parameters to mortality rate. These results suggest that both estimators should be used in combination to determine egg quality. 

Larval length and wet weight were expected to relate to egg quality. However, no larval weight or length showed significant differences among females except for female 5, as opposed to egg quality estimators. This result could be due to the low densities among different batches exhibiting high spawning volume while keeping a low mortality rate, the interference of rearing-related conditions and/or the early preselection in bad quality batches, i.e., removal of the worst larvae from the bad batches would produce few selected larvae with good trait performance, which could be related to the density-dependent growth [61]. In summary, a good egg quality batch is not a sufficient condition to assure optimal hatching success [47,60] or larvae survival.

### 3.2. Variation of Gene Expression throughout Developmental Stages

Since egg quality estimates by traditional morphological traits do not always correlate to the performance of later life stages, some recent studies have focused on molecular genetics to find the basis of quality variance during production [62]. Early embryonic development is influenced by the intrinsic properties of the fertilised egg before the maternal-to-zygotic transition [63] and cytoplasmic mRNAs that are incorporated into the oocyte [53,63,64]. Thus, there is a tight relationship between egg quality and the expression of developmental stage-specific genes [20,25].

The relative expression of seven stage-specific genes showed significant differences among developmental stages. In our study, *ccnb2* was abundant in unfertilised eggs and decreased in gastrula and later during embryonic development in accordance with previous studies [24]. The gene family of cyclin Bs is involved in cell division, cell cycle progression, meiotic maturation of the oocyte and the first embryonic cleavages which can decide the fate of the egg and embryo as “good” or “bad” quality [25,65]. Differential expression of *ccnb2* during early stages shows the influence of parental genes during the first developmental stages, as previously shown [15,25,66]. It is also an essential component of the cytochrome c oxidase complex IV (NADH dehydrogenase), the last enzyme in the mitochondrial electron transport chain which drives oxidative phosphorylation [67]. The *ndufa4l* mRNA is abundant in the oocyte and plays essential roles during the oocyte-to-embryo transition [67]. No expression of the *ndufa4l* gene was observed either in the unfertilised egg or in the zygote, suggesting its activation during blastula, i.e., upon cell division and respiratory chain activation. Somite formation involves the formation of multinucleated cells and myotubes requiring the activation of muscle-specific genes, e.g., troponin, myosin, alfa actin, and parvalbumin. We found an increased expression of *myhc, actc1, acta* and *tnnt3* genes, which are involved in muscle contraction and motility during the early somite stage until hatching. Also, the expression of the *pvalb1* gene, which is related to calcium signalling, was very high during somitogenesis as recently reported for genes related to organogenetic processes [63,68].

### 3.3. Variation of Gene Expression throughout the Spawning Season

Egg quality in Atlantic cod over the spawning season depends at least on fish age, nutritional status, batch number, and stress factors, and the phenomenon of lower egg quality with gamete ageing has been reported in other species such as seabass [69]. Studies on correlating gene expression and egg quality throughout the spawning season are lacking in batch-spawning fishes. In our study, variation of gene expression was higher between spawning batches than among females. Noteworthily, genes *ccnb2, acta, tnnt3* and *pvalb1* showed significant variation in expression among batches and were upregulated after batch 4 for most females analysed. Results show the influence of spawning time on egg quality, despite previous studies indicating that spawning time is of less importance than female parent contribution [67,70]. Gene acta, *tnnt3* and *pvalb1* are reported to be involved in muscle development and regulating muscle contraction in zebrafish and were not expressed until early somitogenesis as we showed in our study [71,72]. 

### 3.4. Embryo Survival in Relation to Gene Expression

Previous studies working on the variation in embryonic mortality and maternal transcript expression have shown that transcript expression of certain genes may be useful for assessing egg quality (e.g., Ref. [73] in halibut). Previous studies in cod lacked a significant correlation between genes and egg quality indicators which makes their use as single biomarkers questionable [74].

Here, we show a positive correlation across females between traditional quality estimators measured at early stages and myofibrillar genes, alpha cardiac muscle (*actc1*) and alpha skeletal muscle (*acta*), expressed during later stages (somite stage and hatched larvae). As per female expression, genes *acta, acta1* and *pvalb1* were also positively correlated with quality estimators and negatively correlated with mortality rate. Despite *acta* and *actc1* expression being measured at later stages than traditional quality variables (FS and NC), results suggest that good traditional quality eggs might lead to the upregulation of both genes during somitogenesis (see Figure 2). New primers for these genes need to be tested in the early stages to corroborate our results since tested primers did not show optimal amplification in the early stages where FS and NC are measured.

Expected “good” quality female batches such as female 11 were mainly responsible for the high significance while “bad” quality families did not show such a correlation. Noteworthily, the expression of both *actc1* and *acta* genes was also shown to differ between sexes in cod broodstock in previous studies, i.e., being downregulated in reproductive males [75]. This suggests that actin function in skeletal muscle cells (e.g., maintenance of the cytoskeleton, cell motility and muscle contraction) could be impaired during the reproductive period and the spawning season. Thus, both genes could be suitable biomarkers for cod egg quality due to their essential role in skeletal muscle development and the swimming capacity of the larvae.

## 4. Materials and Methods

All animal procedures and handling described in this study were carried out according to Norwegian animal welfare laws and were approved by the Norwegian Animal Research Authority (Forsøksdyrutvalget; FOTS ID 11236).

### 4.1. Broodstock Maintenance, Egg Incubation and Larval Rearing

Twelve mature males and twelve mature females of Atlantic cod (*Gadus morhua*) from the sea cage facility of the National Cod Breeding Program at Røsnes, Troms (Centre for Marine Aquaculture—CMA sea cage facility) were transferred to the CMA land-based facility at Kraknes (Norwegian Institute of Food, Fisheries, and Aquaculture Research—NOFIMA, Tromsø, Norway). The broodstock fish were individually tagged using Passive Integrated Transponder (PIT—Sokymat, Switzerland) and maintained in 25 m^3^ tanks at ambient photoperiod, salinity at 34 psu and temperature of 4–6 °C along the spawning season. Prior to the expected spawning dates, egg collectors were installed in the tanks and checked daily. Stripping of gametes was started after the first sign of spawning (presence of eggs in egg collectors) by applying gentle pressure to the abdomen to release ripped eggs. Then, broodstock was checked every 48 h for gametes and four to seven batches per female were achieved during the two-month span of the spring spawning season (Table 6). For traceability purposes, eggs from each female were fertilised with sperm from the same male throughout their reproductive season and the pairing was chosen at random. Eggs stripped from each female were fertilised and incubated regardless its quality as determined by the traditional methods.

#### 4.1.1. Egg collection

Four to seven batches per female were collected during the spawning season depending on the timing required for gamete maturation. A minimum of 100 mL fertilised eggs per batch were incubated in conical silo tanks (25 L, 3.5–5.3 °C, constant aeration, continuous light and continuous upwelling water flow of 1.5 L/min) and egg batches were kept separately throughout incubation. Time was expressed in hours post-fertilisation (hpf) and eggs were classified in development stages based on cell and somite number. Embryos were sampled at six embryonic stages using standard morphological criteria [76], i.e., unfertilised egg (UE), zygote (ZY) at 10 hpf, blastula (BL) at 48 hpf, gastrula (GA) at 96 hpf, early somitogenesis (ES) at 192 hpf, late somitogenesis (LS) at 480 hpf, and newly hatched larvae (HL).

Fertilisation success, cleavage pattern and egg mortality were monitored and recorded after fertilisation. At 20 hpf, an aliquot of cod embryos (about 150 embryos) was sampled from the incubator and viewed under a stereo microscope at 20× magnification, counting the number of unfertilised and fertilised eggs. Fertilisation success was calculated in percentage as the ratio of the number of fertilised to the total number of eggs counted. Among the fertilised eggs, the number of eggs that had abnormal cleavage patterns (asymmetrical cell cleavage of embryos) was also counted [77]. During egg incubation, dead eggs became white and sunk to the bottom and dead eggs were drained out daily from the incubators during the first five days and then every other day until hatching. Fifty percent hatching marked the starting larval age, i.e., 0 days post-hatching (dph; Ref. [28]). Dead egg ratio was estimated by extrapolation of dead egg volume per sample to the total volume. Mortality rate (MR) was estimated as (1). The expected survival rate (SR) was the opposite value to MR.

MR = ((dead egg volume)/(spawn volume)) × 100(1)

#### 4.1.2. Female Selection

Selection of females for forward analysis was based on standard quality measures (Figure 1), i.e., “good-quality” females those showing high FS and NC average values in their batches, while “poor-quality”, i.e., those with low values. Based on this quality criterion [5], we selected all egg batches from five females as representative of bad and good batches (see * in Table 6) to be grown until larvae hatching. The simultaneous tracking of various egg batches was important to observe the variation of egg quality along the spawning season per female as well as among females. Additionally, the number of families was increased to eight to add statistical power to gene expression analyses (see Table 6 indicated by + sign).

#### 4.1.3. Larval Rearing

After 100% of eggs hatched, 3000–15,000 larvae per batch from five females (see Table 6 indicated by * sign) were transferred to 190 L circular fibreglass tanks in replicate (two tanks per batch). Selection of females was based on the quality classification previously mentioned to have a representation of both egg qualities. The number of larvae transferred varied due to variable hatching success in different batches; in some instances, not enough larvae were available for tank replicates. Larvae were reared using standard protocols from CMA facility [78]. Dead larvae were daily removed and counted. Ten larvae from each tank were sampled at 2 dph, 30 dph, 60 dph, and 90 dph, anesthetised with 75 mg/L MS-222 [79] and their standard length was measured using a stereo microscope. Larval length and wet weight from each tank were also recorded at 90 dph.

### 4.2. Gene Expression Analysis

A total of 364 egg, embryo and newly hatched larval samples from eight females (see Table 6 indicated by + sign) were preserved in 1 mL of RNAlater for molecular analyses and stored at −80 °C. Genomic RNA was extracted from homogenates of fifteen embryos per developmental stage and female, and from ten newly hatched larvae per female. Samples were homogenised with a pellet pestle (Kimble, Fisher) in sterile 1.5 mL test tubes containing 1 mL Trizol reagent (NZYol, NZYTech) based on the protocol described by Ref. [80]. The RNA isolates were diluted in RNase-free ddH_2_O and treated with NZY DNase I (NZYTech), followed by enzyme inactivation with 1 V chloroform. RNA integrity and quantity were assessed with a NanoDrop Lite Spectrophotometer (Thermo Scientific) and visualised in 1% agarose gels (NZY DNA Ladder V). All RNA samples were equalised to 300 ng/μL for gene expression comparison up to 8 μL of final volume. Reverse transcription was performed using NZY First-Strand cDNA Synthesis Kit (NZYTech) up to 20 μL of final volume.

Target genes were selected from published DNA sequences based on their role during embryogenesis [22,25,75,81,82,83,84,85,86] in addition to the Atlantic cod genome draft (gadMor1) available from the Ensembl software 112 (https://www.ensembl.org), in July 2018 (European Bioinformatics Institute, Cambridge, UK). This draft was used to identify gene sequences and to develop primer sets using both, Primer3Plus [40] and NCBI Primer-Blast [41]. Gene amplification of all target genes was tested in all developmental stages but not all values for specific stages were reported due to the suboptimal efficiency. Based on optimal amplification results, the relative expression of seven target genes was analysed using quantitative real-time PCR (qPCR) (Table 3; Figure S4) out of fifty candidate genes tested (Table S5). Forty-eight well plates were run in a StepOne Real-Time PCR System (Applied Biosystems, Foster City, CA, USA) using 2 μL cDNA from each RT reaction in duplicate, plus 5 μL NZYSpeedy qPCR Green Master Mix (2×) ROX plus (NZYTech), 0.4 μL per primer (10 μM, forward and reverse) and 2.2 μL nuclease-free water to a final reaction volume of 10 µL. For efficiency calculations, each plate included a 1:5 serial dilution standard curve in duplicate, a cDNA mix from all samples, and sample controls. Analyses of qPCR were run under a 2 min activation and a denaturing step at 95 °C, followed by 40 cycles of 95 °C for 5 s at, 30 s annealing (58–62 °C, Table 3) and 30 s at 72 °C for extension. The mean number of cycles required for the fluorescent signal to exceed the background level (CT mean) was calculated per gene as the arithmetic mean of sample duplicates with a standard deviation lower than one unit.

The programs geNorm, BestKeeper and DataAssist [81,87,88] were used to calculate the expression efficiency (E) and stability of four potential housekeeping or reference genes (Table 3). Stability results were similar among programs and indicated that *rps9* and *ubi* genes had lower standard deviations overall (S.D. = 0.32 and S.D. = 1.32, respectively, Table S4). These genes were taken as reference genes together with *hsp90β*, which was expressed across all developmental stages. The relative quantity of gene expression was calculated with the comparative CT method [80,83,89]. The CT was employed to calculate the relative gene expression and was normalised with the selected reference genes. A selected reference sample formed with the arithmetic mean of all CT values from each analytical group, i.e., either development stages or spawning batches.

### 4.3. Statistical Analysis

A three-way ANOVA analysis was performed to compare standard quality estimators after Kolmogorov–Smirnov test of normality (FS *p*-value = 0.61; NC *p*-value = 0.069). Significant differences in any global test (*p* < 0.05) were followed by pairwise Tukey post hoc test. Testing for Pearson correlations (R) and regression values (R2) between morphological estimators of egg quality was performed with R v3.6.3. software (https://www.r-project.org). Regarding genetic data, two levels of comparison were explored using ANOVA to provide standard deviations and *p*-values on the relative expression of stage-specific genes: (1) comparison among development stages and (2) comparison among spawning batches. The Pearson correlation (r) coefficient between phenotypic (morphological) estimators and molecular data was computed with R software.

## 5. Conclusions

Our study gives a new approach to the egg quality determinants during embryonic development in Atlantic cod. We have tested whether standard egg quality measures such as fertilisation success and normal cleavage were correlated with up/downregulation of stage-specific gene expression profiles (unfertilised egg to hatched larvae). As expected, results revealed the differential expression of several genes during the critical transition from maternal to zygotic transcription. In the present study, we tested the possibility of incorporating genomic techniques such as gene expression profiles of cod eggs, as quality predictors of subsequent life stages, due to the positive significant correlation observed between quality estimators based on egg morphology and the later genetic expression of genes *acta* and *acta1* during somitogenesis. The inclusion of molecular profiles may not be directly used by the aquaculture industry because of the cost and time needed to do the molecular analysis. However, understanding the molecular basis of egg quality may enhance and fine tune morphological selection criteria. This may also provide useful information to improve the broodstock quality because the initial embryonic development is governed by the maternally derived RNA during which time the embryonic mortality is high. Further molecular characterisation of those genes in the broodstock would allow the incorporation of their genotypic information into spawning protocols and breeding programs to improve survival. This knowledge will eventually become beneficial to the aquaculture industry.

## Figures and Tables

**Table 1:** Analysis of variance of fertilisation success (FS, %) and normal cleavage (NC, %) comprising all spawns per female. Spawn number (SN); standard deviation (S.D.); standard error of the mean (S.E.); *p* value (*p*). Pairwise post hoc probabilities after Tukey’s test indicated as Q statistic values.

	SN	Mean	S.D.	S.E.	*p*	Tukey HSD—Q Statistic										
FS (%)						F2	F3	F4	F5	F6	F7	F8	F9	F10	F11	F12
Female 1	7	64.13	13.55	5.12	<0.001	2.13	3.81	0.79	5.48 *	3.27	2.36	3.92	2.58	1.19	1.06	0.58
Female 2	6	47.85	29.52	12.05			1.53	1.07	3.14	1.10	4.12	1.64	0.35	3.10	1.09	1.37
Female 3	7	36.13	27.29	10.32				2.46	1.67	0.39	5.60 **	0.11	1.23	4.66	2.72	2.87
Female 4	4	57.35	17.84	8.92					3.89	2.06	2.79	2.56	1.41	1.77	0.12	0.22
Female 5	7	23.81	18.00	6.80						2.00	7.03 **	1.56	2.90	6.19 **	4.38	4.39
Female 6	6	39.10	21.11	8.62							5.11 *	0.50	0.79	4.15	2.23	2.41
Female 7	4	84.45	3.040	1.52								5.70 **	4.56	1.17	3.24	2.66
Female 8	7	35.30	17.09	6.46									1.34	4.76 *	2.83	2.97
Female 9	7	45.17	14.43	5.45										3.54	1.50	1.75
Female 10	5	73.68	14.53	6.50											2.12	1.60
Female 11	7	56.29	17.90	6.77												0.27
Female 12	6	59.40	26.78	10.93												
NC (%)																
Female 1	7	52.61	15.43	5.83	<0.001	2.19	3.00	1.56	4.58	3.62	3.21	4.71 *	2.99	1.62	0.70	0.06
Female 2	6	34.50	30.08	12.28			0.69	0.37	2.21	1.38	5.01 *	2.33	0.68	3.58	1.50	2.00
Female 3	7	28.79	27.83	10.52				1.00	1.58	0.74	5.77 **	1.71	0.02	4.36	2.27	2.76
Female 4	4	38.03	21.50	10.75					2.34	1.60	4.23	2.45	0.98	2.88	0.95	1.43
Female 5	7	16.33	19.45	7.35						0.78	7.12 **	0.13	1.59	5.80 **	3.83	4.24
Female 6	6	22.72	26.99	11.02							6.24 **	0.90	0.75	4.90 *	2.92	3.35
Female 7	4	82.28	4.49	2.25								7.23 **	5.76 *	1.58	3.77	3.10
Female 8	7	15.30	3.13	1.18									1.72	5.92 **	3.96	4.36
Female 9	7	28.91	20.14	7.61										4.35	2.26	2.74
Female 10	5	66.56	18.90	8.45											2.25	1.59
Female 11	7	46.96	21.61	8.17												0.59
Female 12	6	51.98	24.09	9.83												

*, *p* < 0.5; **, *p* < 0.001.

**Table 2:** Spawning properties and egg quality indicators of five females whose offspring were transferred to first feeding tanks. Length and wet weight are given for larvae reared until 90 dph.

	Spawn Volume	MR	FS	NC	Length t90	Weight t90
Female 1	261.71 ± 166.53	4.76	64.13 ± 13.55	52.61 ± 15.43	36.23 ± 3.33	0.40 ± 0.13
Female 5	226.14 ± 103.40	5.94	23.81 ± 18.00	16.33 ± 19.45	40.51 ± 7.61	0.65 ± 0.29
Female 6	171.50 ± 81.22	8.18	39.10 ± 21.11	22.72 ± 26.99	37.07 ± 3.52	0.50 ± 0.14
Female 7	207.00 ± 109.18	2.34	84.45 ± 3.04	82.28 ± 4.49	37.04 ± 4.86	0.55 ± 0.21
Female 11	209.43 ± 124.22	5.39	56.29 ± 17.90	46.96 ± 21.61	32.66 ± 4.25	0.36 ± 0.18

**Table 3:** Selected target genes and reference genes for expression analyses. Code, gene abbreviation name; function, known gene function; stages, developmental stages that showed optimal amplification, i.e., unfertilised egg (UE), zygote (ZY), blastula (BL), gastrula (GA), early somitogenesis (ES), late somitogenesis (LS) and newly hatched larva (HL); PCR primer sequences (5′-3′); size, PCR product size in base pairs; Tm, annealing temperature in °C; E, qPCR efficiency. Novel primers for genes *ndufa4l*, *tnnt3* and *pvalb1* were developed using Primer3Plus software, v3.3.0 (https://www.primer3plus.com/index.html, accessed on 20 May 2019) [40] and NCBI Primer-Blast (https://www.ncbi.nlm.nih.gov/tools/primer-blast/index.cgi, accessed on 20 May 2019) [41].

Gene	Code	Function	Stages	5′-Forward Primer-3′	5′-Reverse Primer-3′	Size	Tm	E
Target genes								
Cyclin B2	*ccnb2*	Mitosis regulation	UE-GA	GGCCGGTAGTGCACCATGGC	TCAGGAGAGCCTCAAAGGCTGCA	106	60	92.86
Myosin	*myhc*	Muscle contraction	ES-HL	CAGAAGCTATAAAAGGTGTCCG	GCAGCCATTCTTCTTATCCTCCTC	86	60	101.8
Actin alpha cardiac muscle 1	*actc1*	Muscle contraction	ES-HL	CTTCCCTGTCCACCTTCCAG	ACGGAGACGACGATGGAGAA	122	62	97.88
Actin alpha skeletal muscle	*acta*	Muscle contraction	LS-HL	TGTTCACAGTTCGTTCTCCGA	TCGTCTCCGTCGTCATCATC	200	62	86.04
NADH ubiquinone reductase 4l	*ndufa4l*	Respiratory chain	BL-GA	CTTTTTCATCGGTGGAGGCG	TTGTTCTTGCGATCCCAGCT	90	60	97.22
Troponin T skeletal	*tnnt3*	Muscle contraction	LS-HL	ACATGGGCTCCAACTACAGC	TTGCGTCTTCCAGCCAGAAT	112	62	96.97
Parvalbumin 1	*pvalb1*	Calcium signalling	LS-HL	CAGAGCGGCTTCATTGAGGA	CTCCGATCATGCCATCACCA	136	62	83.21
Reference genes								
Ribosomal protein S9	*rps9*	40S ribosomal protein	BL-HL	TCTTTGAAGGTAATGCTCTGTTGAGA	CGAGGATGTAATCCAACTTCATCTT	84	62	85.26
Acidic ribosomal protein	*arp*	60 S ribosomal protein	BL-HL	TGATCCTCCACGACGATGAG	CAGGGCCTTGGCGAAGA	113	62	87.11
Ubiquitin	*ubi*	Protein transport and degradation	BL-HL	GGCCGCAAAGATGCAGAT	CTGGGCTCGACCTCAAGAGT	69	58	98.32
Heat Shock Protein 90-Beta	*hsp90β*	Chaperone, polypeptides stabilisation	UE-HL	CGTGGCGTGGTGGACTCT	GACTATGTTCTTGCGGATGACCTT	96	58	94.45

**Table 4:** Correlation (Pearson coefficient of correlation, R) matrix among standard quality indicators (fertilisation success—FS, normal cleavage—NC and mortality rate—MR) and target genes using average data (all females). Genetic data used were average of all developmental stages (see stages under analysis in Table 3) for each spawning batch.

	FS	NC	MR	*acta*	*actc1*	*ccnb2*	*myhc*	*ndufa4l*	*pvalb1*	*tnnt3*
FS	-									
NC	0.912 *	-								
MR	−0.336	−0.379	-							
*acta*	0.290 *	0.295 *	−0.167	-						
*actc1*	0.307 *	0.262	−0.161	0.592	-					
*ccnb2*	0.090	0.094	0.281	0.117	−0.085	-				
*myhc*	−0.002	0.066	−0.153	0.529 *	0.360 *	−0.017	-			
*ndufa4l*	0.123	0.197	0.024	0.148	−0.074	−0.088	−0.029	-		
*pvalb1*	0.211	0.156	−0.353	0.589 *	0.612 *	−0.114	0.272	0.138	-	
*tnnt3*	−0.044	−0.048	0.049	0.313	0.464	−0.017	0.308	−0.139	0.387 *	-

*, significant values (*p* < 0.05).

**Table 5:** Correlation (Pearson coefficient of correlation, R) matrix among standard quality indicators (fertilisation success—FS, normal cleavage—NC and mortality rate—MR) and target genes using average data (all females). Genetic data used were average of all developmental stages (see stages under analysis in Table 3) for each spawning batch.

	F1	F3	F5	F6	F7	F8	F9	F11
Fertilisation success								
FS/NC	0.919 *	0.984 *	0.984 *	0.786	0.991 *	0.744 *	0.931 *	0.865 *
FS/MR	−0.219	−0.394	0.417	−0.187	−0.568	−0.997	0.344	−0.954 *
FS/*acta*	0.487	0.319	0.777 *	0.684	0.908	0.334	−0.065	0.717
FS/*actc1*	0.280	0.876*	0.565	0.457	0.881	0.757 *	0.240	0.799 *
FS/*ccnb2*	0.658	−0.135	0.674	0.401	0.609	−0.417	−0.078	−0.236
FS/*myhc*	−0.050	−0.310	−0.010	−0.091	0.879	0.431	0.358	0.482
FS/*ndufa4l*	−0.126	0.052	−0.353	0.588	−0.805	−0.090	0.053	0.029
FS/*pvalb1*	−0.738 *	0.698	0.198	0.304	0.825	0.664	−0.648	0.739
FS/*tnnt3*	0.003	−0.476	0.472	0.271	0.697	0.395	−0.462	0.691
Normal cleavage								
NC/MR	−0.073	−0.248	0.316	−0.283	−0.666	−0.738	0.195	−0.929 *
NC/*acta*	0.222	0.206	0.780 *	0.911 *	0.870	0.689	0.229	0.715
NC/*actc1*	0.076	0.797 *	0.541	0.661	0.876	0.498	0.429	0.772 *
NC/*ccnb2*	0.563 *	−0.059	0.596	0.116	0.500	−0.122	0.111	−0.480
NC/*myhc*	−0.075	−0.380	−0.089	0.440	0.834	0.732	0.598	0.546
NC/*ndufa4l*	0.064	−0.024	−0.358	0.936 *	−0.731	0.203	0.152	−0.098
NC/*pvalb1*	−0.662	0.607	0.148	0.589	0.743	0.523	−0.405	0.521
NC/*tnnt3*	0.002	−0.413	0.377	0.120	0.607	0.291	−0.186	0.762 *
Mortality rate								
MR/*acta*	−0.637	−0.402	−0.054	−0.125	−0.248	−0.021	−0.191	−0.626
MR/*actc1*	−0.661	−0.561	0.035	0.150	−0.400	−0.703	−0.213	−0.795
MR/*ccnb2*	0.208	0.375	0.637	−0.599	0.291	0.709	−0.150	0.464
MR/*myhc*	−0.796 *	−0.276	−0.114	−0.026	−0.181	−0.286	0.328	−0.383
MR/*ndufa4l*	0.834 *	−0.245	0.286	−0.124	0.129	−0.157	0.014	0.137
MR/*pvalb1*	−0.179	−0.536	−0.430	−0.446	−0.023	−0.541	−0.710	−0.794
MR/*tnnt3*	−0.417	0.624	0.107	−0.476	0.188	−0.118	0.567	−0.653

*, significant values (*p* < 0.05).

**Table 6:** Sampling details of the 12 cod females analysed. The number of samples comprises seven embryonic stages per egg batch.

Female	No. Batches	No. Samples
Female 1 *^,+^	7	49
Female 2	6	42
Female 3 ^+^	7	49
Female 4	4	28
Female 5 *^,+^	7	49
Female 6 *^,+^	6	42
Female 7 *^,+^	4	28
Female 8 ^+^	7	49
Female 9 ^+^	7	49
Female 10	5	35
Female 11 *^,+^	7	49
Female 12	6	42

*, females whose larvae were transferred to first feeding tanks and reared until 90 dph; ^+^, females whose eggs were genetically analysed with qPCR.

## Data Availability

Data are contained within the article and Supplementary Materials.

## Supplementary Materials

The supporting information can be downloaded at: https://www.mdpi.com/article/10.3390/ijms25137488/s1.
