# Supplementary material for: Gene Expression and Phenotypic Assessment of Egg Quality across Developmental Stages of Atlantic Cod throughout the Spawning Season
Source: Int J Mol Sci. 2024 Jul 8;25(13):7488. doi: 10.3390/ijms25137488 (PMC11242223; doi:10.3390/ijms25137488)
Supplement: Supplementary file 1 [file ijms-25-07488-s001.zip › ijms-3020019-supplementary.pdf]

Supplementary Materials

Figure S1. Variation in fertilisation success (FS %) and normal cleavage (NC %) between spawning batches (1 to 7, see also Table 1) within female cod.

Figure S2: Pearson correlation (R) and linear regression (R2) of egg quality variation from 11 female cod. FS (%), average fertilisation success; NC (%), normal cleavage; MR, (%), mortality rate; L (cm), larval length at t0 and t90 (dph, days post hatch).

Table S1: Mortality rate per female calculated as the average proportion of dead egg volume (ml, $\bar{x} \pm$ S.D.) to the egg spawn volume (ml, $\bar{x} \pm$ S.D.). Expected survival rate (%) per female was calculated as the opposite value to the mortality rate (%).

Figure S3: Linear plot of larval length (cm) among batches within the five selected females, at t0, t30, t60 and t90 dph.

Table S2: Variation of larval length at 0, 30, 60 and 90 dph (cm, t0, t30, t60, t90) in four to seven spawning batches (S1 - S7) per female. Average length ($\bar{x}$), variance ($\sigma 2$), standard deviation (σ) and ANOVA test p-value (p) among females within larvae age as obtained with global ANOVA. *, all larvae died; **, no data available

Table S3: Wet weight variation among 90 dph larvae from potential good-quality-egg females (females 6 and 7) and poor-quality egg females (females 1 and 5) in four to seven spawning batches (S1 - S7). Average weight ($\bar{x}$) variance ($\sigma 2$) and *p*-value (*p*) obtained by one-factor ANOVA are also indicated. *all larvae died, **no data available.

Figure S4: Dissociation curves of seven target genes (A-G), i.e. cyclin B2, *ccnb2*; myosin, *myhc*; actin, alpha cardiac muscle 1, *actc1*; actin alpha skeletal muscle, *acta*; NADH ubiquinone reductase 4l, *ndufa4l*; troponin T skeletal, *tnnt3*; parvalbumin 1, *pvalb1*; and four reference genes (H-K), i.e., ribosomal protein S9, *rps9*; acidic ribosomal protein, *arp*; ubiquitin, *ubi*; heat Shock Protein 90-Beta, *hsp90β*.

Figure S5. Cycle threshold values (CT) for expression levels of seven target genes (a) and four reference genes (b) (see gene codes in Table 3). CT values are given as median (lines), 25th to 75th percentile (boxes) and range (whiskers). Samples belonged to seven embryonic stages and four to seven egg spawning batches.

Table S4. Expression stability value of potential reference genes during the embryonic development of Atlantic cod using software geNorm (M-value < 1.5), BestKeeper (S.D.) and DataAssist (Score). In bold, best stability values per gene and algorithm.

Table S5. Gene name (Name), known function (Function), primer sequences (5′- 3′), product size (Size, bp) and annealing temperature (Tm, ºC) of candidate genes tested. Novel gene primers were developed using Primer3Plus software [40] and NCBI Primer-Blast [41].

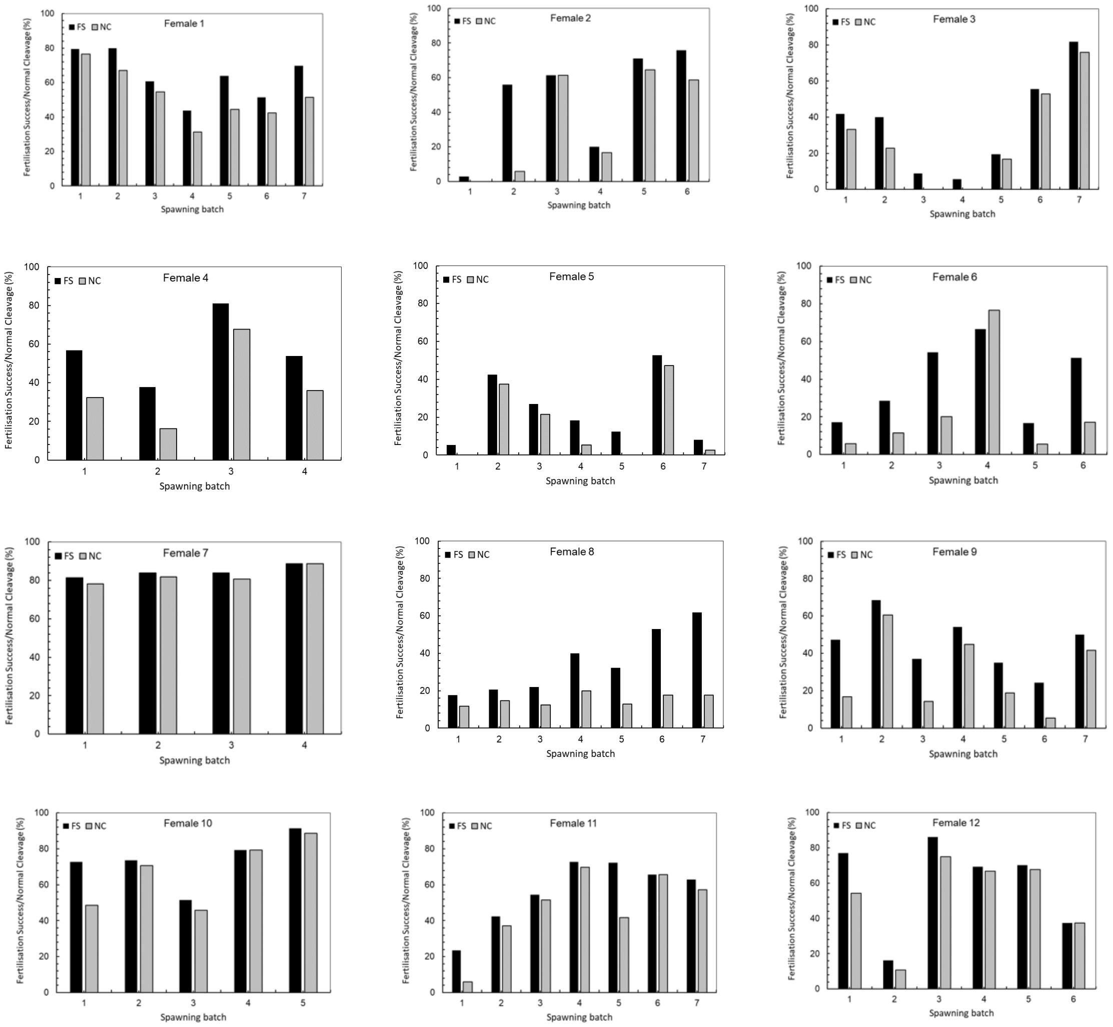

Figure S1. Variation in fertilisation success (FS %) and normal cleavage (NC %) between spawning batches (1 to 7, see also Table 1) within female cod.

Table S1. Mortality rate per female calculated as the average proportion of dead egg volume (ml, $\bar{x} \pm$ S.D.) to the egg spawn volume (ml, $\bar{x} \pm$ S.D.). Expected survival rate (%) per female was calculated as the opposite value to the mortality rate (%).

	Spawn volume	Dead egg volume	Mortality rate	Survival rate
Female 1	261.71±166.53	12.46± 7.63	4.76	95.24
Female 2	156.33±77.54	11.00±5.97	7.04	92.97
Female 3	240.29±149.10	17.86±12.93	7.43	92.57
Female 4*	450.50±100.03	42.23±8.59*	9.37	90.63
Female 5	226.14±103.40	13.43±8.85	5.94	94.06
Female 6	171.50±81.22	13.94±11.68	8.18	91.87
Female 7	207.00±109.18	4.85±3.88	2.34	97.66
Female 8	165.00±160.65	12.17±9.82	7.38	92.62
Female 9	186.43±123.58	11.51±7.35	6.18	93.82
Female 10	199.40±57.22	11.94±4.04	5.99	94.01
Female 11	209.43±124.22	11.28±6.77	5.39	94.61
Female 12	199.33±140.35	13.22±15.98	6.63	93.37

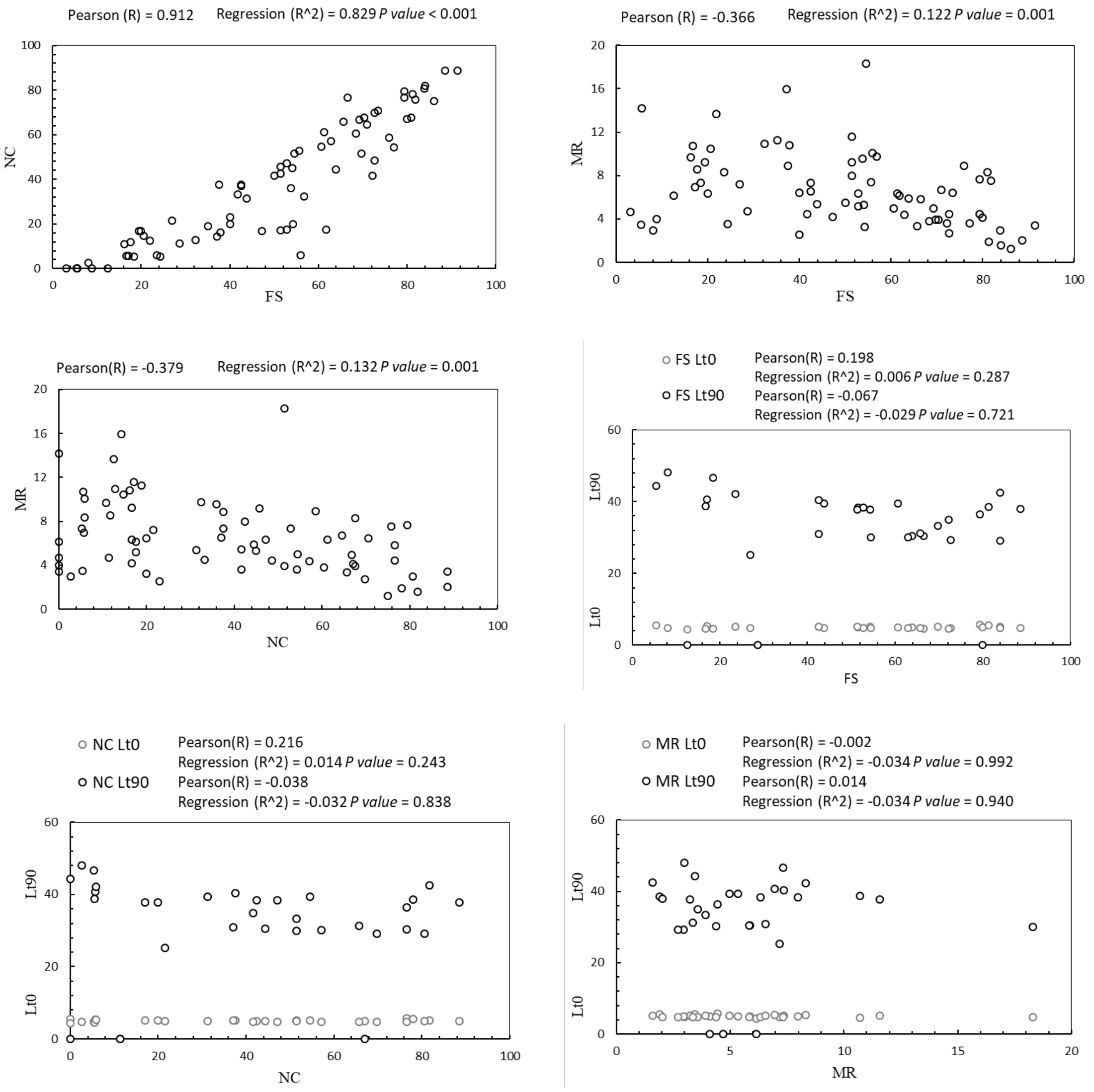

Figure S2. Pearson correlation (R) and linear regression (R2) of egg quality variation from 11 female cod. FS (%), average fertilisation success; NC (%), normal cleavage; MR, (%), mortality rate; L (cm), larval length at t0 and t90 (dph, days post hatch).

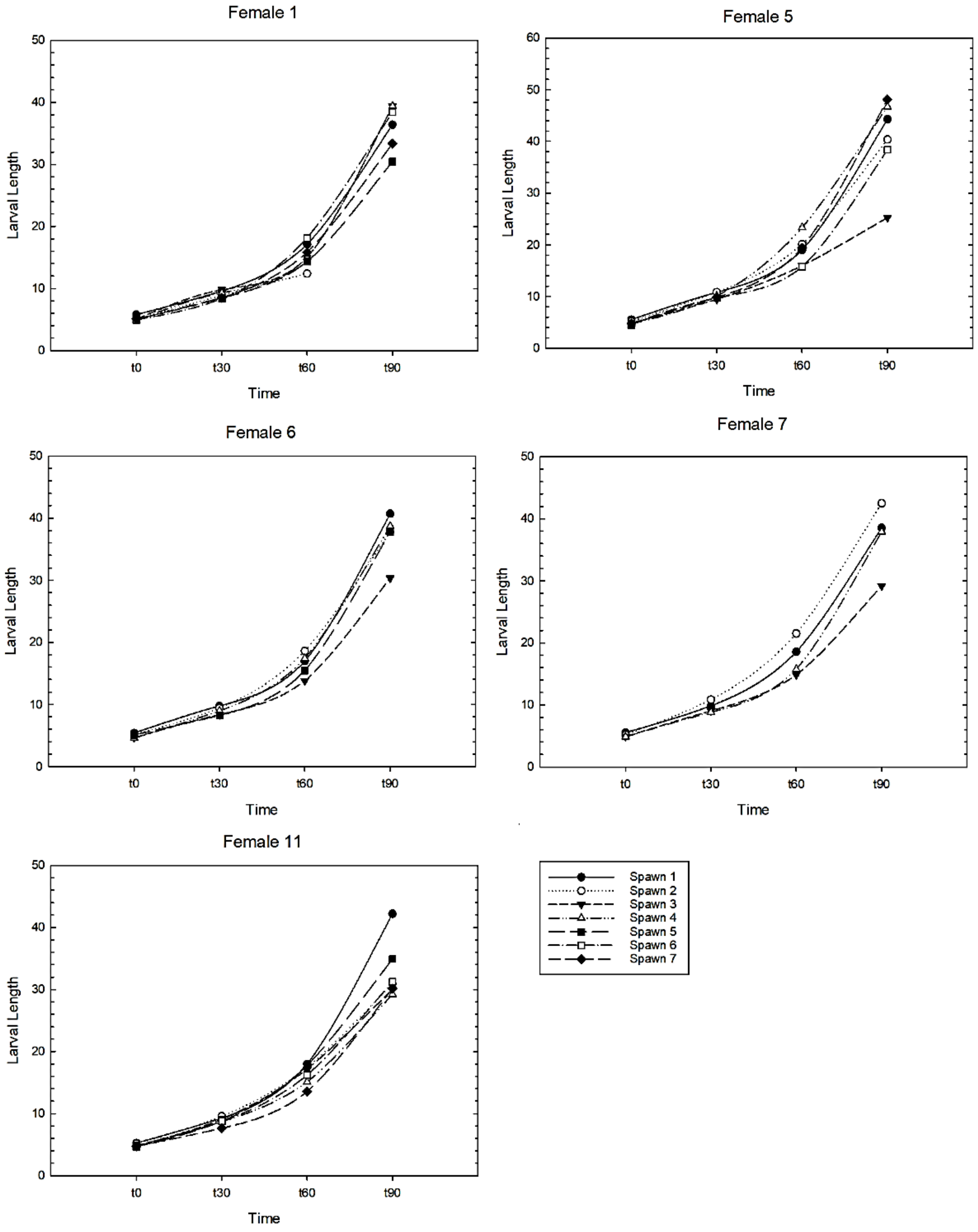

Figure S3. Linear plot of larval length (cm) among batches within the five selected females, at t0, t30, t60 and t90 dph.

Table S2. Variation of larval length at 0, 30, 60 and 90 dph (cm, t0, t30, t60, t90) in four to seven spawning batches (S1 - S7) per female. Average length ($\bar{x}$), variance ($\sigma 2$), standard deviation (σ) and ANOVA test p-value (*p*) among females within larvae age as obtained with global ANOVA. *, all larvae died; **, no data available.

	Larval Length (cm)										
Larval age	S1	S2	S3	S4	S5	S6	S7	$\bar{x}$	$\sigma 2$	σ	*p*
t_0											
Female 1	5.78	5.02	5.06	4.86	4.97	4.96	5.10	5.11	0.08	0.28	0.51
Female 5	5.53	5.16	4.85	4.68	4.41	4.74	4.73	4.87	0.12	0.33	
Female 6	5.39	*	5.19	4.67	4.59	5.09	**	4.99	0.09	0.31	
Female 7	5.53	5.17	4.89	4.85	**	**	**	5.11	0.07	0.27	
Female 11	5.26	5.15	4.83	4.76	4.67	4.78	4.73	4.88	0.04	0.21	
t30											
Female 1	9.58	8.88	9.80	9.28	8.48	8.36	8.51	8.98	0.28	0.53	0.01*
Female 5	10.79	10.83	9.47	10.11	*	9.73	9.84	10.13	0.26	0.51	
Female 6	9.75	*	9.40	8.36	8.97	8.24	**	8.94	0.34	0.58	
Female 7	9.85	10.85	9.03	8.80	**	**	**	9.63	0.64	0.80	
Female 11	9.28	9.56	9.10	8.70	8.77	8.74	7.64	8.83	0.32	0.56	
t60											
Female 1	17.11	12.39	14.96	14.96	14.32	18.14	15.83	15.39	3.03	1.74	0.09
Female 5	18.97	20.11	15.94	23.30	*	15.72	19.42	18.91	6.66	2.58	
Female 6	16.97	*	18.60	13.80	17.41	15.46	**	16.45	2.76	1.66	
Female 7	18.55	21.50	14.84	15.73	**	**	**	17.66	6.80	2.60	
Female 11	17.97	17.44	17.17	15.10	17.74	16.20	13.54	16.45	2.26	1.50	
t90											
Female 1	36.40	*	39.40	39.40	30.45	38.40	33.35	36.23	11.09	3.33	0.09
Female 5	44.30	40.35	25.24	46.70	**	38.35	48.10	40.51	58.03	7.61	
Female 6	40.70	*	37.80	30.35	38.70	37.80	**	37.07	12.41	3.52	
Female 7	38.55	42.50	29.19	37.90	**	**	**	37.04	23.61	4.86	
Female 11	42.20	30.91	30.00	29.23	34.90	31.25	30.15	32.66	18.03	4.25	

Table S3. Wet weight variation among 90 dph larvae from potential good-quality-egg females (females 6 and 7) and poor-quality egg females (females 1 and 5) in four to seven spawning batches (S1 - S7). Average weight ($\bar{x}$) variance ($\sigma 2$) and p-value (p) obtained by one-factor ANOVA are also indicated. *all larvae died, **no data available.

	Wet weight									
	S1	S2	S3	S4	S5	S6	S7	$\bar{x}$	$\sigma 2$	p
Female 1	0.43	*	0.23	0.57	0.27	0.50	0.38	0.40	0.02	0.126
Female 5	0.76	0.60	0.15	0.90	*	0.54	0.93	0.65	0.08	
Female 6	0.66	*	0.50	0.27	0.55	0.52	**	0.50	0.02	
Female 7	0.56	0.65	0.25	0.73	**	**	**	0.55	0.04	
Female 11	0.74	0.25	0.27	0.25	0.47	0.31	0.26	0.36	0.03	

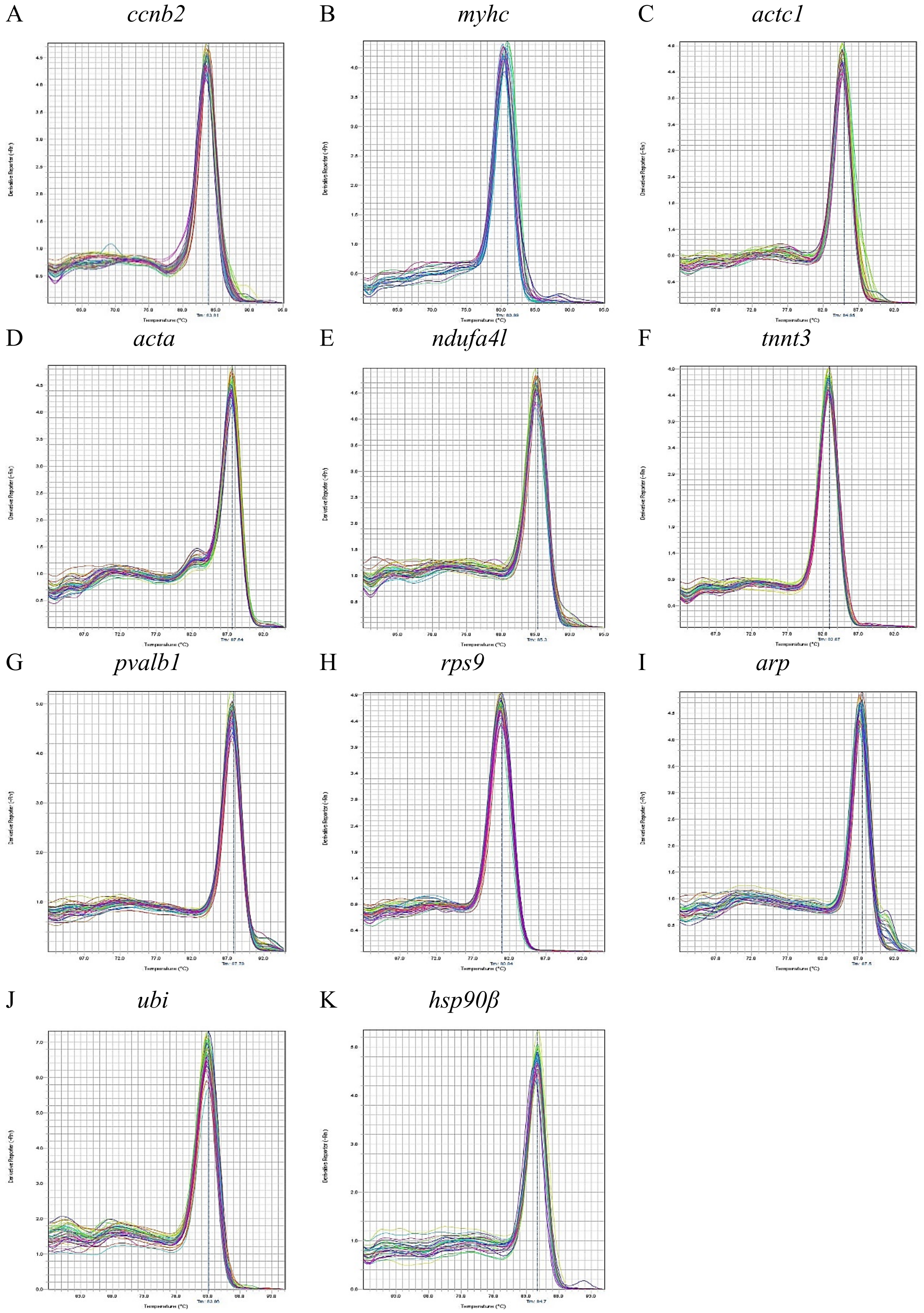

Figure S4. Dissociation curves of seven target genes (A-G), i.e. cyclin B2, *ccnb2*; myosin, *myhc*; actin, alpha cardiac muscle 1, *actc1*; actin alpha skeletal muscle, *acta*; NADH ubiquinone reductase 4l, *ndufa4l*; troponin T skeletal, *tnnt3*; parvalbumin 1, *pvalb1*; and four reference genes (H-K), i.e., ribosomal protein S9, *rps9*; acidic ribosomal protein, *arp*; ubiquitin, *ubi*; heat Shock Protein 90-Beta, *hsp90β*.

a

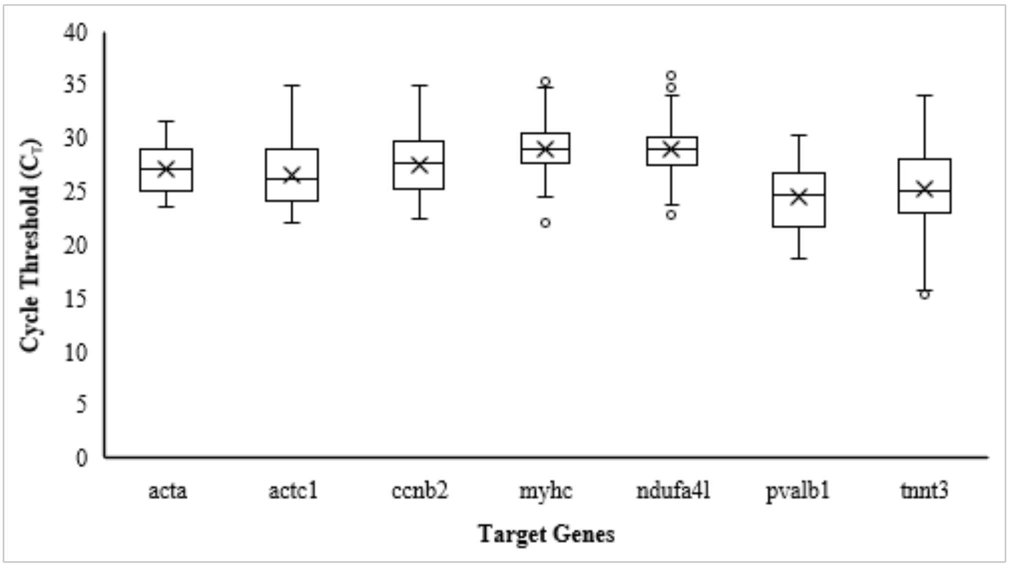

b

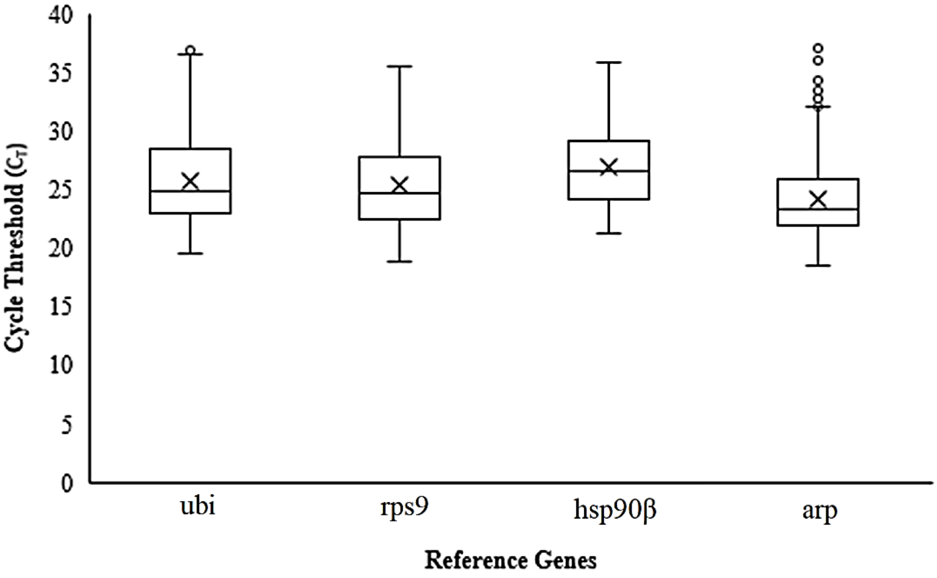

Figure S5. Cycle threshold values (CT) for expression levels of seven target genes (a) and four reference genes (b) (see gene codes in Table 3). CT values are given as median (lines), 25th to 75th percentile (boxes) and range (whiskers). Samples belonged to seven embryonic stages and four to seven egg spawning batches.

Table S4. Expression stability value of potential reference genes during the embryonic development of Atlantic cod using software geNorm (M-value < 1.5), BestKeeper (S.D.) and DataAssist (Score). In bold, best stability values per gene and algorithm.

	rps9	*ubi*	*hsp90β*	*arp*
geNorm				
M-value	**0.11**	**0.12**	0.14	0.16
BestKeeper				
S.D.	**1.32**	**1.32**	1.39	1.43
DataAssist				
Score	**1.69**	**1.78**	2.22	2.22

Table S5. Gene name (Name), known function (Function), primer sequences (5’- 3’), product size (Size, bp) and annealing temperature (Tm, ºC) of candidate genes tested. Novel gene primers were developed using Primer3Plus software [40] and NCBI Primer-Blast [41].

Name	Function	5’-Forward primer-3’	5’-Reverse primer-3’	Size	Tm	Source
Lipovitellin 2	Early metabolism (lipid and metal storage)	TCAGCAAAGCCATTCAGATG	TCGGTCCTCAATCCAATCTC	175	53.30-54	This study
Lipovitellin 2	Early metabolism (lipid and metal storage)	GCCAAGAAGGAGCCCAGATT	CAAGAGCAGTTACCAGGCCA	139	57.56-57.36	This study
Cyclin B2	Cell cycle regulatory machinery	GGCCGGTAGTGCACCATGGC	TCAGGAGAGCCTCAAAGGCTGCA	106	63.59-62.63	[83]
Zona pellucida glycoprotein C	Putative primary sperm receptor	GGGTGTCCTTGGTCACCGTCC	TCTCCTGCGACTCGGCAGCT	110	62.42-63.44	[83]
Myosin 11	Contractile protein	AACTCAGGCGTGGCACCGAG	CTGAGGCGTCGTCCACCACG	90	62.60-62.91	[83]
Tropomyosin 1 alpha chain	Contractile protein	CTTCTCGGCCTGGGCCTCCA	GCACAGAGGAGCGCGCTGAG	113	64.39-63.49	[83]
Zygote arrest 1-like	Oocyte to embryo transition	CCTTCCGTAAACGTGGGTAA	TTGTTCCAGAGATGCACCAG	145	54.63-54.99	This study
Zygote arrest 1-like	Oocyte to embryo transition	GGTCCTTGGAACCGAGAAGG	ATAAGCACTCTCCCACCGGA	145	57.62-57.94	This study
Beta 2 microglobulin	Immune system	CAATCAGACCGACCTGTCCT	GCACGCATACGACTCTTCCT	94	56.52-57.19	This study
Beta 2 microglobulin	Immune system	TCCCCAATGCCAATCAGACC	GTTTGCCGATGTGTGTGACC	126	57.60-56.96	This study
E- Cadherin	Cell reorganization and migration	TCACCTACGGCTTCATCACA	CCCACTGTTGATCCCTCTTG	100	55.92-55.36	This study
E- Cadherin	Cell reorganization and migration	ATGAACCCTGCGACTGGATG	GATGCTGTTGTTCTCCCCCA	149	55.92-55.36	This study
High choriolytic enzyme 1	Hydrolysis of the inner layer of fish egg envelope	TGACTACAGCTCGGTCATGC	CTGGCCAATGGGAATGTAAG	100	56.97-53.69	This study
High choriolytic enzyme 1	Hydrolysis of the inner layer of fish egg envelope	CGTCTCTCCCCTCTACGACA	GACCAGCACCCATATCGAGG	144	57.67-57.49	This study
Nanog Homeobox cell proliferation	Maintaining pluripotency of embryonic stem cells	AGCCCAATCGGGACTCACTT	TTCTTGGCATTGTAGTGCTCAGA	117	59.4-58.9	[22]
Nanor	Ion transmembrane transport	ATCCAATACCCAACGGTTCA	GCGATGAAATGGCTGAATCT	92	55.3	[20]
Cyclin B	Mitosis regulation	CGGGAGATGGAGATGACTGT	TCTCGTAGTCCACCATGCAG	150	59.4	[87]
Cyclin B2	Mitosis regulation	GGCCGGTAGTGCACCATGGC	TCAGGAGAGCCTCAAAGGCTGCA	106	65.5-64.2	[83]

Zona pellucida glycoprotein	Sperm binding and acrosome reaction	GGAGCTCTCTGAGGAGGGAA	GCCTCAAAGATCGGGGGAAA	144	64.1-59.4	[83]
Zona pellucida glycoprotein C	Sperm binding and acrosome reaction	GGGTGTCCTTGGTCACCGTCC	TCTCCTGCGACTCGGCAGCT	110	65.7-63.5	[83]
beta-2-microglobulin	Immune system	GAGCCCAACACCCTGATCT	GCTCGATGGTGATCTCTGG	61	58.8	[88]
Beta-2-microglobulin	Immune system	CTTCGACAAGACCTGGCACTTC	TCCGTTTGCCGATGTGTGT	-	62.1-56.7	[89]
Myosin	Muscle contraction	CAGAAGCTATAAAAGGTGTCCG	GCAGCCATTCTTCTTATCCTCCTC	86	658.4-62.7	[82i]
Myosin heavy chain	Muscle contraction	ATGTAACGGTGTGCTGGAGG	GTACTCGTCGTGAGGCACAT	187	59.4	[75]
Myosin light chain 2	Muscle contraction	GGACGTGCTGGCCTCTATGG	GGATCCGCACCCTTCAGCTT	135	63.5-61.4	[75]
Myosin heavy polypeptide 11	Muscle contraction	GCGGACACATCAGTACACGACTGG	GTCGGTCCACCATGTCGGCC	128	66.1-65.5	[83]
Actin, alpha cardiac muscle 1	Muscle contraction	CTTCCCTGTCCACCTTCCAG	ACGGAGACGACGATGGAGAA	122	61.4-59.4	[75]
Actin, alpha skeletal muscle	Muscle contraction	TGTTCACAGTTCGTTCTCCGA	TCGTCTCCGTCGTCATCATC	200	57.9-59.4	[75]
Lipovitellin 3 (vitellogenin)	Early metabolism (lipid and metal storage)	GCCACAGAGTTCCTGATGCT	AAGTCGCCAGTGAATCCAGG	173	59.4	This study
Zygote arrest (arrestin 3a, retinal)	Oocyte to gamete transition	TCGGGAGCAGGAAAGTGTTC	TCTTCATCAGGGTGTCGTGC	171	61.4-59.4	This study
NADH ubiquinone reductase 4l	Respiratory-chain	CTTTTTCATCGGTGGAGGCG	TTGTTCTTGCGATCCCAGCT	90	59.4-57.3	This study
NADH ubiquinone reductase 9a	Respiratory-chain	GGAGTACATCTACGCCGTGG	GAACTTCAGGTCCGTCGTGT	157	61.4-59.4	This study
Cyclin B1	Mitosis regulation	TGCTGGGACATGAAGTGACC	GGTCCTGAAGAAAGCGGTCA	135	59.4	This study
Cyclin B2	Mitosis regulation	AGGTGTGACGGCTATGTTGG	TTGCAGTATCTGGGGCTTGG	112	59.4	This study
Zona pellucida-like domain containing 1a	Sperm binding and acrosome reaction	CGCGGCTTCATCAACAACAA	ACAGGTTCCCATAGGCGTTG	130	57.3-59.4	This study
Zona pellucida-like domain containing 1b	Sperm binding and acrosome reaction	CAACTCTCAGGTGGCGTCTT	GTGGGGATGGACAGAGGTTG	126	59.4-61.4	This study
Cadherin 24	Cell adhesion	TGCAGGCTAAAGACATGGGG	GAGTCAGACGCCGAGATACG	178	59.4-61.4	This study
Muscle creatine kinase	Muscle ATP consumption	ATCATCTCCGTGTGATCGCC	CACATGAAGCCGTGGTTGTG	121	59.4	This study
Troponin T skeletal	Muscle contraction	ACATGGGCTCCAACTACAGC	TTGCGTCTTCCAGCCAGAAT	112	59.4-57.3	This study
Parvalbumin 1	Calcium signaling.	CAGAGCGGCTTCATTGAGGA	CTCCGATCATGCCATCACCA	136	59.4	This study
Crystallin Gamma S	Transparency and refractive index of the lens	CACTCCTATCTGAGCCGCTG	GTACTGGGACCCGCTTGAAA	192	61.4-59.4	This study

Astacin, metallo-endopeptidase	Extracellular proteins and polypeptides degradation	TGGTCACCATCGTCACCTTG	GTTCTCGATAAGGCGGGAGG	128	59.4-61.4	This study
ikaros	Chromatin remodeling	ATGATCTCCGGGTCTGTGAG	ACACTTGAGCTTTCCGTCGT	165	60	[20]
Ribosomal protein S9	40S ribosomal protein	TCTTTGAAGGTAATGCTCTGTTGAGA	CGAGGATGTAATCCAACTTCATCTT	84	56.13-54.77	[81]
Acidic ribosomal protein	60 S ribosomal protein	TGATCCTCCACGACGATGAG	CAGGGCCTTGGCGAAGA	113	56.22-58.08	[81]
Ubiquitin	Protein degradation via the proteasome	GGCCGCAAAGATGCAGAT	CTGGGCTCGACCTCAAGAGT	69	56.25-58.85	[81]
Heat Shock Protein 90-Beta	Chaperone, polypeptides stabilization and fold	CGTGGCGTGGTGGACTCT	GACTATGTTCTTGCGGATGACCTT	96	60	[22]
Elongation factor 1 α	Protein synthesis	CCCTGTGGAAGTGGCTGAAG	CATCCAAGGGTCCGTATCTCTT	93	60	[22]
β-actin	Cytoskeletal structural protein	CACAGCCGAGCGTGAGATT	ACGAGCTAGAAGCGGTTTGC	95	60	[22]
Glyceraldehyde-3-phosphate dehydrogenase	Glycolytic protein	CCATGACAACTTTGGCATCGT	AGGGTCCGTCCACTGTCTTCT	83	60	[22]
